# Activated mesothelial cells produce heparin-binding growth factors: implications for tumour metastases

**DOI:** 10.1054/bjoc.1999.1068

**Published:** 2000-02-21

**Authors:** D G Jayne, S L Perry, E Morrison, S M Farmery, P J Guillou

**Affiliations:** Surgical Unit, Level 8, Clinical Sciences Building, St James's University Hospital, Beckett Street, Leeds, LS9 7TF, UK

**Keywords:** peritoneum, mesothelial cell, cytokine, heparin-binding growth factors, tumour metastasis

## Abstract

Curative surgery for gastrointestinal malignancy is commonly thwarted by local tumour recurrence. The heparin-binding growth factors, basic fibroblast growth factor (bFGF), heparin-binding epidermal growth factor-like growth factor (HB-EGF) and vascular epidermal growth factor (VEGF) are all implicated in the metastatic process, but whether or not these essential growth factors are produced by the activated peritoneum is unknown. This study reveals that peritoneal mesothelial cells constitutively express mRNA for bFGF, HB-EGF and two VEGF spliced variants, VEGF_121_and VEGF_165_. Mesothelial activation with interleukin (IL)-1b or tumour necrosis factor (TNF)-a produced an up-regulation of mRNA for HB-EGF and VEGF, but not bFGF expression. IL-6 failed to stimulate growth factor expression, whereas IL-2 produced a marked suppression in HB-EGF and bFGF, but not VEGF expression. Mesothelial cells were shown to predominantly express mRNA for the intermediate affinity (bg_c_) IL-2 receptor. Cytokine-induced growth factor up-regulation was confirmed at the protein level using Western blotting of mesothelial cell lysates for HB-EGF and culture supernatant enzyme-linked immunosorbent assay for VEGF. The production of these growth factors by human mesothelial cells may play a significant role in post-operative peritoneal tumour recurrence. Their common heparin-binding property offers a potential therapeutic target for manipulating the growth factor environment of the human peritoneum. © 2000 Cancer Research Campaign


*British Journal of Cancer* (2000) **82**(6), 1233–1238
© 2000 Cancer Research Campaign
Article no. bjoc.1999.1068, available online at http://www.idealibrary.com on IDEAL®

### Activated mesothelial cells produce heparin-binding growth factors: implications for tumour metastases

**DG Jayne, SL Perry, E Morrison, SM Farmery and PJ Guillou**

Professorial Surgical Unit, Level 8, Clinical Sciences Building, St James's University Hospital, Beckett Street, Leeds LS9 7TF, UK

**Summary** Curative surgery for gastrointestinal malignancy is commonly thwarted by local tumour recurrence. The heparin-binding growth factors, basic fibroblast growth factor (bFGF), heparin-binding epidermal growth factor-like growth factor (HB-EGF) and vascular epidermal growth factor (VEGF) are all implicated in the metastatic process, but whether or not these essential growth factors are produced by the activated peritoneum is unknown. This study reveals that peritoneal mesothelial cells constitutively express mRNA for bFGF, HB-EGF and two VEGF spliced variants, $VEGF_{121}$ and $VEGF_{165}$. Mesothelial activation with interleukin (IL)-1b or tumour necrosis factor (TNF)-a produced an up-regulation of mRNA for HB-EGF and VEGF, but not bFGF expression. IL-6 failed to stimulate growth factor expression, whereas IL-2 produced a marked suppression in HB-EGF and bFGF, but not VEGF expression. Mesothelial cells were shown to predominantly express mRNA for the intermediate affinity ($bg_c$) IL-2 receptor. Cytokine-induced growth factor up-regulation was confirmed at the protein level using Western blotting of mesothelial cell lysates for HB-EGF and culture supernatant enzyme-linked immunosorbent assay for VEGF. The production of these growth factors by human mesothelial cells may play a significant role in post-operative peritoneal tumour recurrence. Their common heparin-binding property offers a potential therapeutic target for manipulating the growth factor environment of the human peritoneum. © 2000 Cancer Research Campaign

**Keywords:** peritoneum; mesothelial cell; cytokine; heparin-binding growth factors; tumour metastasis

Curative resection for gastrointestinal cancer often fails because of local tumour recurrence. This is a catastrophic event for the patient and a substantial problem for the clinician, as it is seldom amenable to further surgical resection. Strategies that reduce the incidence of this complication should improve the prognosis of these common malignancies.

The peritoneum is the host tissue for tumour cells disseminated within the abdominal cavity, and is composed of an inner mesothelial cell monolayer supported on a connective tissue matrix (diZerega and Rodgers, 1993). Surgical exploration traumatizes this peritoneal lining, leading to the synthesis of a cytokine-rich inflammatory exudate (Badia et al, 1996). Interleukin-1b (IL-1b), tumour necrosis factor-a (TNF-a) and interleukin-6 (IL-6) are present early in the inflammatory response, and stimulate the local production of growth factors that promote peritoneal repair and healing (Fukasawa et al, 1989). This growth factor-rich environment may provide a favourable milieu for the establishment of secondary tumour metastases (Radinsky, 1993).

Although little is known about the growth factors produced by the human peritoneum in response to trauma, basic fibroblast growth factor (bFGF), heparin-binding epidermal growth factor-like growth factor (HB-EGF) and vascular epidermal growth factor (VEGF) are ubiquitously expressed in healing wounds. They share a common heparin-binding ability which is critical to their function, enabling them to bind to heparan-sulphate proteoglycans on cell surfaces and in the extracellular matrix. Unlike bFGF and HB-EGF, several different isoforms of VEGF are produced by alternate mRNA splicing (Houck et al, 1991), which differ in their heparin-binding properties: $VEGF_{206}$ and $VEGF_{189}$ show a high, $VEGF_{165}$ an intermediate, and $VEGF_{121}$ a low affinity for heparin.

In addition to their role in wound healing, HB-EGF, bFGF and VEGF are also implicated in the metastatic process. HB-EGF is a ligand for the EGF receptor (EGFR), which is frequently up-regulated in gastrointestinal tumours, and may have a role as a host-derived paracrine growth factor (Miyoshi et al, 1997). bFGF is an important growth factor in the gastrointestinal tract and may act as an autocrine growth factor in oesophageal cancer (Iida et al, 1994). VEGF acts synergistically with bFGF, augmenting tumour cell metastasis by up-regulating endothelial adhesion molecules (Pepper et al, 1992). In addition, the $VEGF_{165}$ variant may exert a direct effect on tumour cell behaviour by binding of its heparin-binding domain to tumour cell receptors (Soker et al, 1996, 1998). All three growth factors, HB-EGF, bFGF and VEGF, have angiogenic properties.

Given their diverse attributes, it may be hypothesized that the production of these growth factors by the surgically injured peritoneum may play a significant role in local tumour recurrence. The aim of the current study was to determine whether peritoneal mesothelial cells produce bFGF, HB-EGF and VEGF and to determine the effect of cytokine stimulation on growth factor expression. IL-1b, TNF-a and IL-6 were chosen as representative cytokines generated in the peritoneal inflammatory response. The T-cell-associated cytokine, IL-2, was studied for comparison, as it was thought unlikely to produce any effect.

*Received 1 July 1999*
*Revised 12 October 1999*
*Accepted 12 October 1999*

*Correspondence to:* D Jayne

### MATERIALS AND METHODS

### Mesothelial cell culture and characterization

Samples of greater omentum were obtained from patients undergoing elective abdominal surgery (local ethical committee approved) and subjected to enzymatic disaggregation, as described previously (Stylianou et al, 1990). Briefly, omental samples were cut into approximately 10-$cm^2$ pieces, washed in phosphate-buffered saline (PBS) and incubated in HBSS supplemented with 0.1% glucose (w/v), penicillin–streptomycin (50 IU $ml^{-1}$, 50 mg $ml^{-1}$), and 0.25% trypsin (v/v) for 20 min at 37 C. Following incubation, samples were centrifuged at 100 ***g*** for 5 min and the omentum and supernatant discarded. The cell pellet was resuspended in Ham's F12 culture medium supplemented with 10% fetal calf serum (FCS), penicillin–streptomycin (50 IU $ml^{-1}$, 50 mg $ml^{-1}$), insulin (0.1 IU $ml^{-1}$) and holo-transferrin (5 mg $ml^{-1}$, Sigma, Poole, UK) and seeded at 1 ¥ $10^5$ cells $ml^{-1}$ into 75-$cm^2$ tissue culture flasks. This complete medium was used throughout, apart form the induction of resting cultures and in stimulation experiments when a reduced medium of Ham's F12 supplemented with 1% FCS was substituted. All cultures were incubated at 37 C, 5% carbon dioxide/95% oxygen, and the media changed every 3 days.

Primary cultures were characterized by indirect fluorescent immunocytochemistry. Representative cells were grown to confluence on 1% gelatin (w/v)-coated glass slides, washed in PBS, and fixed with 4% paraformaldehyde (pH 7.4) for 10 min at 4 C. Slides were blocked with 10% rabbit serum (Dako, Buckinghamshire, UK) and incubated for 1 h at room temperature with mouse primary antibodies directed against human cytokeratin 8, 18, vimentin, BerEp4, von Willebrand factor (Dako) and calretinin (Chemicon, Harrow, UK). Following 3 washes in PBS, slides were incubated with a fluorescein isothiocyanate (FITC)-labelled rabbit anti-mouse secondary antibody for 1 h at room temperature, washed in PBS and visualized using a Leitz™ fluorescent microscope.

### Cytokine stimulation, RNA extraction and semi-quantitative RT-PCR

Primary mesothelial cultures were passaged with 0.25% trypsin–EDTA (v/v), seeded into gelatin-coated 24-well culture dishes at 1 ¥ $10^5$ cells $well^{-1}$ and grown to confluence. Confluent cultures were rested for 24 h in reduced medium prior to stimulation. Reduced medium alone or supplemented with physiological doses (0.1, 0.2, 0.5, 1.0, 2.0 ng $ml^{-1}$) of IL-1b, TNF-a, IL-6, or IL-2 (R&D Systems, Oxon, UK) was used to challenge the cultures for differing time points. To exclude extraneous sources of growth factor stimulation, additional experiments were performed using reduced medium supplemented with cytokine carrier protein (0.1% bovine serum albumin (BSA), Sigma), or endotoxin (lipopolysaccharide, 10 ng $ml^{-1}$, Sigma).

Total RNA was extracted using the cationic detergent Catrimox™ (VH Bio Ltd, Newcastle-upon-Tyne, UK) and 10-ml reverse transcribed with oligo $dT_{15}$ (0.5 mg), MMLV-RT (Promega, Southampton, UK, 120 U), dNTPs (1 mM each), RNAsin (Promega, 20 U), magnesium chloride ($MgCl_2$) (2 mM) to a total volume of 20 ml at 42 C for 55 min and inactivated at 95 C for 5 min. cDNA was amplified in multiplex reactions using pre-aliquoted polymerase chain reaction (PCR) master mix tubes (Advanced Biotechnologies, Surrey, UK) containing 1 mM primer, 0.625 U *Taq* DNA Polymerase, 75 mM Tris–HCl, 20 mM $(NH_4)_2SO_4$, 2 mM $MgCl_2$, 0.01% (v/v) Tween-20 and 0.2 mM dNTPs to a total volume of 25 ml. PCR was performed for 35 cycles of 1 min at 95 C, 1 min at 55 C, 1 min at 72 C and a final elongation step of 5 min at 72 C. Gene-specific primers were designed to span at least one intron and titrated against the house-keeper gene, glyceraldehyde-3-phosphodehydrogenase (GAPDH), for multiplex PCR. The following growth factor primer sets were used: bFGF: forward CTGTACTGCAAAAACGGG, reverse AAAGTATAGCTTTCTGCCA; HB-EGF: forward TTATCCTC-CAAGCCACAAGC, reverse ACAGATGACAGCACCACAGC; VEGF(total): forward CTACCTCCACCATGCCAAGT, reverse ATGTTGGACTCCTCAGTGGG; VEGF(spliced variants): forward GCACAACAAATGTGAATGCAG, reverse TGGTGAGAGATC-TGGTTCCC; GAPDH: forward GAGTCAACGGATTTGGTCGT, reverse TTCCCGTTCTCAGCCTTGAC. All experiments included control reactions excluding the template or using untranscribed RNA. Amplified products were separated on 2% agarose gels and visualized by ethidium bromide ultraviolet fluorescence. Semi-quantitative analysis was performed using a digital image analysis system (Gelbase™, UV Products, Cambridge, UK) Growth factor expression was recorded relative to that of GAPDH. VEGF-spliced variants identified by primers spanning the splicing site (exons 4 and 8) were purified using a cDNA extraction kit (Qiagen, Surrey, UK) and sequenced by Oswel Laboratories Ltd, Southampton, UK.

### Identification of mesothelial IL-2 receptor message by RT-PCR

Mesothelial cells were grown to confluence in 24-well culture dishes, and rested in reduced medium for 24 h. Cultures were challenged with reduced medium alone or supplemented with cytokine (1 ng $ml^{-1}$ IL-1b or TNF). Total RNA was extracted from unstimulated and cytokine activated cells after 4-h incubation, and subjected to reverse transcription PCR (RT-PCR) as described above. Gene-specific primers for the a-, b- and g-chains of the IL-2 receptor were: a-chain: forward ATGGGAAAATGAA GCCACAG, reverse GACGAGGCAGGAAGTCTCAC; b-chain: forward TCTCCCTCCAAGTTGTCCAC, reverse TCA-GGACCTTCTTCAGCCAT; g-chain: forward GTGCTCAG-CATTGGAGTGAA, reverse ACTGACGAGGCAGAGTCGTT. Mononuclear cells from normal subjects were isolated by standard Ficol separation (Lymphoprep™, Nycomed Pharma, Birmingham, UK), and activated by incubation with 2 mg/ml phytohaemagglutinin (PHA, Sigma) in RPMI plus 10% FCS culture medium for 3 days, and used as positive controls for IL-2 receptor expression.

### Western blotting of mesothelial cell lysates for HB-EGF

Mesothelial cells were grown to confluence in gelatin-coated 25-$cm^2$ tissue culture flasks, and rested in reduced medium for 24 h prior to stimulation with 1 ng $ml^{-1}$ of cytokine. Cells were scraped free, washed in PBS and boiled in RIPA/Laemmli (Laemmli, 1970) buffer (1:2 v/v) containing b-mercaptoethanol (1:20, v/v) for 5 min. Cell lysates were electrophoresed on 18% polyacrylamide gels, transferred to nitrocellulose membranes, blocked with 1% dried milk power in PBS (w/v) and stained with goat anti-human HB-EGF polyclonal antibody (1:200, R&D Systems)

 *© 2000 Cancer Research Campaign*

or mouse anti-human cytokeratin 18 antibody as a loading control (1:500, Dako). After washing in PBS, membranes were incubated with an appropriate horseradish peroxidase (HRP)-labelled secondary antibody and visualized by enhanced chemiluminescence (Supersignal™, Pierce & Warriner, Chester, UK) on Hyperfilm™ (Amersham Life Sciences, Buckinghamshire, UK)

### Mesothelial cell conditioned media ELISA

Mesothelial cells were grown to confluence in gelatin-coated 25-$cm^2$ tissue culture flasks and rested in reduced medium for 24 h. Cultures were stimulated with 1 ng $ml^{-1}$ of cytokine in 5 ml of reduced medium for a further 24 h. Control (unconditioned) cytokine supplemented media were incubated in identical culture flasks under the same conditions. Both mesothelial conditioned and unconditioned media were centrifuged at 100 ***g*** for 5 min. Analysis of VEGF in the supernatants was performed using standard ELISA methodology (R&D Systems) according to the manufacturer's instructions.

### RESULTS

### Mesothelial culture and characterization

Primary mesothelial cells grew to form confluent cultures with a characteristic cobblestone appearance. Immunocytochemistry detected strong intracellular staining for cytokeratin 8, 18, vimentin and calretinin, confirming the mesothelial phenotype and excluding fibroblast culture. Contamination by endothelial and epithelial cells was excluded by negative staining for von Willebrand factor and BerEp4 surface antigen respectively.

### Growth factor analysis by semi-quantitative RT-PCR

Resting mesothelial cultures constitutively expressed bFGF, HB-EGF and VEGF mRNA. The early inflammatory cytokines IL-1b and TNF-a produced a significant dose- and time-dependent increase in HB-EGF (Figure 1A) and total VEGF (Figure 1B) levels compared to control unstimulated samples. This effect was maximal at 4 h post-stimulation and after treatment with 1 ng $ml^{-1}$ of cytokine (Figure 2 A, B). Neither BSA nor endotoxin affected mesothelial growth factor expression within the time course of the experiments, excluding these as a source of growth factor stimulation. IL-6 produced only marginal up-regulation in HB-EGF and VEGF expression within the 8-h time period of the experiments (Figure 2C). None of these cytokines had an effect on mesothelial bFGF expression.

The T-cell associated cytokine, IL-2, was found to have a pronounced suppressive effect on HB-EGF and bFGF expression (Figure 2D) but little effect on VEGF expression. Suppression was maximal 8 h post-stimulation, and subsequently mRNA levels returned towards resting values.

Further analysis of mesothelial VEGF expression revealed the presence of two spliced variants. Both variants were up-regulated in response to IL-1b and TNF-a stimulation (Figure 1C). Direct

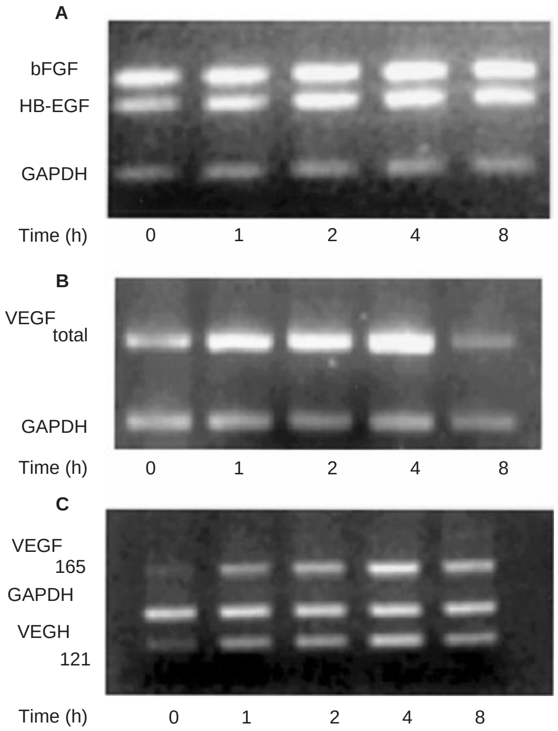

**Figure 1** Representative electrophoretic gels showing the effect of cytokine stimulation on mesothelial growth factor expression. (**A**) Up-regulation of HB-EGF but constant bFGF expression, (**B**) up-regulation of total VEGF expression, (**C**) up-regulation of spliced variants $VEGF_{121}$ and $VEGF_{165}$. GAPDH = housekeeper gene

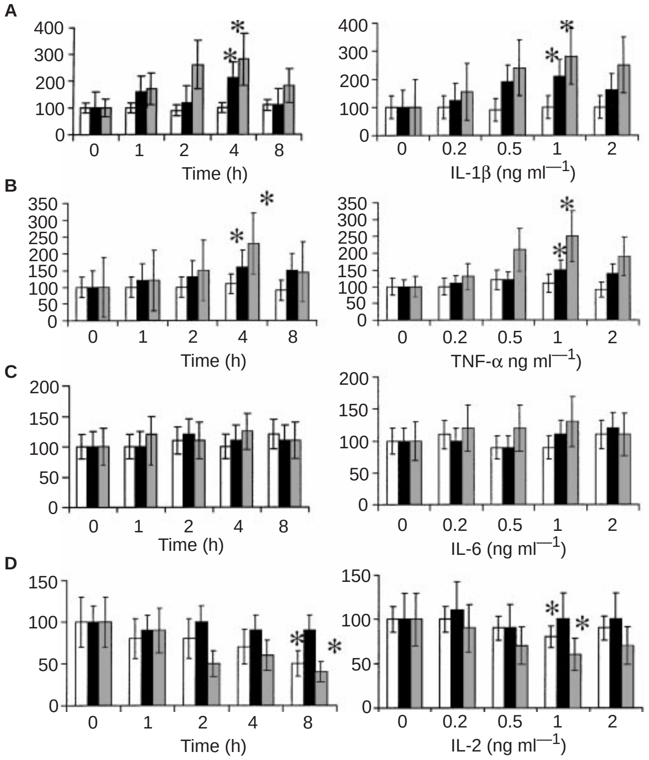

**Figure 2** Semi-quantitative analysis of the effect of cytokine stimulation on resting mesothelial growth factor expression. Values are the median of at least 6 experiments, normalized for controls (100%). (**A**) IL-1b, (**B**) TNF-a, (**C**) IL-6, (**D**) IL-2. *$P < 0.05$ relative to controls, Mann–Whitney *U*-test. ☐ bFGF; ■ VEGF; ▒ HB-EGF

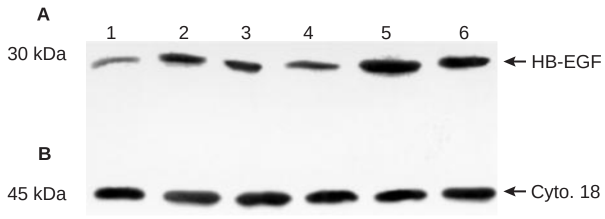

**Figure 3** (**A**) Western blot of mesothelial cell lysates showing effect of cytokine stimulation on HB-EGF production. Lanes 1, 2 and 3 after 4 h stimulation; lanes 4, 5 and 6 after 8 h. Lanes 1 and 4 = unstimulated; lanes 2 and 5 = IL-1β (1 ng ml$^{-1}$); lanes 3 and 6 = TNF-α (1 ng ml$^{-1}$). (**B**) Constant expression of the loading control, cytokeratin 18 (cyto. 18)

sequencing identified these variants as the intermediate heparin binding $VEGF_{165}$ and the non-heparin binding $VEGF_{121}$.

### Expression of mesothelial IL-2 receptor by RT-PCR

As anticipated, PHA activated T-cells showed strong expression of all three components (α-, β-, $γ_c$-chains) of the high affinity IL-2 receptor. By contrast, resting mesothelial cultures showed strong expression of the β- and $γ_c$-chains with only weak expression of the α-chain, indicating the intermediate-affinity (β/$γ_c$) IL-2 receptor as the predominant expressed form (data not shown). Interestingly, mesothelial activation with 1 ng ml$^{-1}$ IL-1β or TNF-α resulted in complete loss of IL-2 receptor message.

### Western blotting for mesothelial HB-EGF protein

Western blotting of mesothelial cell lysates was used to confirm cytokine-induced up-regulation at the protein level. Figure 3 shows the effect of 1 ng ml$^{-1}$ IL-1β and TNF-α on mesothelial HB-EGF protein production, compared to unstimulated cells. Under reducing conditions, a single 22 kDa band was detected in both resting and cytokine activated cultures. In agreement with the mRNA results, both IL-1β and TNF-α up-regulate HB-EGF, but this was most pronounced following IL-1β stimulation. Up-regulation was detectable after 4 h of cytokine stimulation and maintained at 8 h.

### Estimation of mesothelial VEGF production by ELISA

RT-PCR analysis had shown mesothelial VEGF expression to consist of the intermediate and non-membrane binding isoforms, $VEGF_{165}$ and $VEGF_{121}$ respectively. Standard ELISA methods were therefore used to quantify VEGF secretion in conditioned media. Mesothelial cells incubated in reduced medium showed constitutive expression of VEGF protein (0.8 ng ml$^{-1}$) following 24 h incubation (Figure 4). This was significantly increased following incubation with IL-1β or TNF-α supplemented media (1.5 ± 0.1 and 1.3 ± 0.1 ng ml$^{-1}$ respectively). IL-6 and IL-2 had no effect on VEGF secretion compared to control samples.

### DISCUSSION

Mesothelial cells form the innermost layer of the human peritoneum, where they secrete a variety of surfactant-like molecules that aid visceral motility and prevent adhesion formation (Chailley-Heu et al, 1997). Recently, attention has focused on their role in the peritoneal inflammatory response, both as a source of cytokine production and as an interactive surface for cellular defence mechanisms (Topley et al, 1996). They are responsive to the early inflammatory cytokines and under the influence of IL-1β, TNF-α or bacterial haptens, they secrete a variety of cytokines (IL-1α, IL-1β, IL-6), growth factors (GM-CSF, G-CSF, M-CSF) (Lanfrancone et al, 1992) and chemokines (IL-8, RANTES, MCP-1) (Kinnaert et al, 1996) that are integral to peritoneal inflammation. They share similar properties with endothelial cells, and express the adhesion molecules ICAM-1, VCAM-1 and PECAM-1 (Klein et al, 1995) which facilitate lymphocyte trafficking into the peritoneal cavity. However, the role of mesothelial cells and their growth factors in peritoneal repair is less well established. In particular, the production of heparin-binding growth factors such as bFGF, HB-EGF and VEGF by activated human mesothelial cells has not previously been explored.

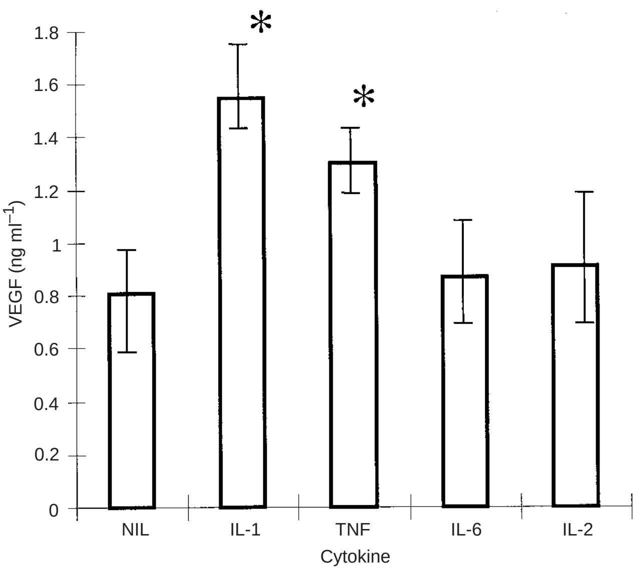

**Figure 4** The effect of cytokine stimulation on mesothelial VEGF production. Rested confluent mesothelial cells were incubated for 24 h in reduced media alone or supplemented with 1 ng ml$^{-1}$ of cytokine and VEGF production determined by ELISA. Values are the medians of 6 experiments, shown with interquartile ranges. **P* < 0.05 relative to controls (Mann–Whitney *U*-test)

These experiments have shown for the first time that concentrations of IL-1β and TNF-α, achieved after surgical operations on the peritoneum, stimulate the production of HB-EGF and VEGF in resting human mesothelial cell cultures. Maximal up-regulation occurred within 4 h of stimulation, suggesting a role for these growth factors in the early peritoneal inflammatory response. Such a role is conceivable for VEGF, previously known as vascular permeability factor (VPF), but the biological role of HB-EGF in this context is less obvious. Of interest is the similar dose and time responses displayed by HB-EGF and VEGF when subjected to cytokine stimulation, and it is interesting to speculate that they may share a common mesothelial regulatory mechanism.

Somewhat surprisingly, IL-2 produced a marked suppression in HB-EGF and bFGF expression in confluent cultures and may play a role in maintaining mesothelial cells in the resting state, as loss

 *© 2000 Cancer Research Campaign*

of IL-2 receptor mRNA occurred upon activation with IL-1$\beta$ or TNF-$\alpha$. The source of IL-2 in the resting peritoneum has not been explored, but an autocrine mechanism is unlikely as IL-2 transcripts have not been detected in long-term mesothelial cultures (Lanfrancone et al, 1992). Investigation of the IL-2 receptor status in resting mesothelial cells revealed strong expression of mRNA for the $\beta$- and $\gamma_c$-chains that constitute the intermediate-affinity ($\beta\gamma_c$) binding receptor. The $\gamma_c$-chain is the major signalling component of the IL-2 receptor, but is common to other cytokine receptors (IL-4, IL-7 and IL-13) (Farner et al, 1997). Indeed, IL-13 has been shown to up-regulate mesothelial VCMA-1 (but not ICAM-1) expression (Sironi et al, 1994), although the effect of IL-4 and IL-7 on mesothelial function has not yet been reported. Interestingly, IL-2 has previously been investigated as a potential adjuvant in intraperitoneal immunotherapy and, in a murine model, the combined injection of lymphokine activated killer (LAK) cells and IL-2 produced a significant reduction in intraperitoneal tumour mass and prolonged survival (Ottow et al, 1987). However, when tumour inoculation was preceded by laparotomy, the therapeutic effects of IL-2 and LAK cell therapy were completely abrogated, with excessive tumour growth at sites of peritoneal trauma (Eggermont et al, 1988). In light of our current findings, part of the anti-tumour effect attributed to IL-2 may be due to its suppressive effect on resting mesothelial growth factor production.

The role of heparin-binding growth factors, such as bFGF, HB-EGF and VEGF, in peritoneal metastasis formation remains to be fully elucidated. HB-EGF is a ligand for the EGFR, which is frequently up-regulated in gastrointestinal tumours, and correlates with tumour aggressiveness and prognosis (Radinsky et al, 1995; Tokunaga et al, 1995). We have previously demonstrated the importance of this growth factor receptor in pancreatic cancer growth (Davies et al, 1993; Gillespie et al, 1993), and others have shown a mitogenic effect of HB-EGF on human gastric (Naef et al, 1996) and pancreatic cancer cell lines (Kobrin et al, 1994). It is envisaged that the production of HB-EGF by the human peritoneum may act as a host-derived growth factor for disseminated gastrointestinal tumour cells. In addition to its proliferative effects, HB-EGF may contribute to other aspects of the metastatic process. It has been shown to promote tumour cell adhesion in human oesophageal (Sato et al, 1996) and breast cancer cells (Narita et al, 1996) by stimulating cellular integrin expression. A similar mechanism is envisaged for the adhesion of disseminated gastrointestinal tumour cells to the activated peritoneum. In in vitro models, HB-EGF promotes endothelial tubule formation in type I collagen gels (Ushiro et al, 1996), and its production by activated mesothelial cells may play a role in peritoneal angiogenesis.

bFGF stimulates the proliferation of several intestinal epithelial cell lines (Dignas et al, 1994) and is an important growth factor in the gastrointestinal tract. Human oesophageal cancer cells have been shown to produce mRNA for bFGF and its receptor, FGFR1, suggesting an autocrine role in oesophageal cancer (Iida et al, 1994). Although we failed to demonstrate a cytokine-induced stimulation of mesothelial bFGF, the cellular trauma associated with surgery is probably the main mechanism by which cytosolic bFGF is released (D'Amore, 1990; Ku and D'Amore, 1995). In this way, bFGF is free to stimulate fibroblast proliferation, interact with metastatic tumour cells and induce angiogenesis (Montesano, 1992).

The role of VEGF in peritoneal tumour metastasis is not confined to angiogenesis. In combination with bFGF, it up-regulates the expression of the endothelial adhesion molecules, VCAM-1 and ICAM-1 (Melder et al, 1996), and the $VEGF_{165}$ variant exerts a direct effect on cell migration and morphology by interaction of its heparin-binding domain with tumour cell receptors (Soker et al, 1996).

The significance of these growth factors for counteracting peritoneal metastasis lies in their heparin-binding ability. This property is essential to their biological action, enabling them to bind to heparan-sulphate proteoglycans on the cell membrane and extracellular matrix (Rapraeger, 1991, 1994). The purpose of this interaction is believed to be multifold; preventing molecular diffusion, providing a potential extracellular growth factor store, and initiating high affinity ligand–receptor interaction. The addition of exogenous heparin disrupts this proteoglycan association, and in the human colon cancer cell line, Caco-2, inhibits bFGF-induced growth and migration responses (Jayson and Gallagher, 1997). In the murine model, the concomitant administration of heparin to mice inoculated with intra-abdominal tumour cells reduces the formation of metastasis at sites of peritoneal trauma (Goldstein et al, 1993).

We have demonstrated that HB-EGF, bFGF and VEGF, are produced by human mesothelial cells and their expression is significantly up-regulated by culture in a cytokine-rich environment. Such an environment exists within the peritoneal cavity immediately after surgical intervention, when free cancer cells may be spilled during the course of the resection. By selectively targeting their common heparin-binding affinity, it should be possible to substantially alter the growth factor environment of the traumatized peritoneum, making it less favourable for metastatic growth. In this way, the infusion of heparin-like solutions in the post-operative period may provide an effective adjuvant to reducing the incidence of local tumour recurrence.

### ACKNOWLEDGEMENTS

This work was undertaken by the Leeds Teaching Hospitals NHS Trust who received funding from the NHS Executive and Yorkshire Cancer Research.

### REFERENCES

Badia JM, Whawell SA, Scott-Coombes DM, Abel PD, Williamson RC and Thompson JN (1996) Peritoneal and systemic cytokine response to laparotomy. *Br J Surg* **83**: 347–348

Chailley-Heu B, Rubio S, Rougier JP, Ducroc R, Barlier-Mur AM, Ronco P and Bourbon JR (1997) Expression of hydrophilic surfactant proteins by mesentery cells in rat and man. *Biochem J* **328**: 251–256

D'Amore PA (1990) Modes of FGF release in vivo and in vitro. *Cancer Metastasis Rev* **9**: 227–238

Davies N, Kapur P, Gillespie J, Guillou PJ and Poston GJ (1993) Transforming growth factor alpha is trophic to pancreatic cancer in vivo. *Gut* **34**: 1097–1098

Dignas AU, Tsunekawa A and Podolsky DK (1994) Fibroblast growth factors modulate intestinal epithelial cell growth and migration. *Gastroenterology* **106**: 1254–1262

diZerega GS and Rodgers KE (1993) Peritoneum. In: *The Peritoneum*, pp. 1–25, Springler-Verlag: New York

Eggermont AM, Steller EP, Marquet RL, Jeekel J and Sugarbaker H (1988) Local regional promotion of tumour growth after abdominal surgery is dominant over immunotherapy with interleukin-2 and lymphokine activated killer cells. *Cancer Detect Prev* **12**: 421–429

*© 2000 Cancer Research Campaign*

Farner NL, Hank JA and Sondel PM (1997) Interleukin-2: molecular and clinical aspects. In: *Cytokines in Health and Disease*, Renwick DG and Friedland JS (eds) Marcel Dekker: New York
Fukasawa M, Yanangihara DL, Rodgers KE and diZerega GS (1989) The mitogenic activity of peritoneal tissue repair cells: control by growth factors. *J Surg Res* **47**: 45–51
Gillespie J, Dye JF, Schachter M and Guillou PJ (1993) Inhibition of pancreatic cancer cell growth in vitro by the tyrphostin group of tyrosine kinase inhibitors. *Br J Cancer* **68**: 1122–1126
Goldstein DS, Lu ML, Hattori T, Ratliff TL, Loughlin KR and Kavoussi LR (1993) Inhibition of peritoneal tumour-cell implantation: model for laparoscopic cancer surgery. *J Endocrinol* **7**: 237–241
Houck KA, Ferrara N, Winer J, Cachianes G, Li B and Leung DW (1991) The vascular endothelial growth factor family – identification of a fourth molecular species and characterization of alternative splicing of RNA. *Mol Endocrinol* **5**: 1806–1814
Iida S, Katoh O, Tokunaga A and Terada M (1994) Expression of the fibroblast growth factor gene family and its receptor gene family in the human upper gastrointestinal tract. *Biochem Biophys Res Commun* **199**: 1113–1119
Jayson GC and Gallagher JT (1997) Heparin oligosaccharides: inhibitors of the biological activity of bFGF on Caco-2 cells. *Br J Cancer* **75**: 9–16
Kinnaert P, De Wilde JP, Bournonville B, Husson C and Salmon I (1996) Direct activation of human peritoneal mesothelial cells by heat-killed microorganisms. *Ann Surg* **224**: 749–755
Klein CL, Bittinger F, Skarke CC, Wagner M, Kohler H, Walgenbach S and Kirkpatrick CJ (1995) Effects of cytokines on the expression of cell adhesion molecules by cultured human omental mesothelial cells. *Pathobiology* **63**: 204–212
Kobrin MS, Funatomi H, Friess H, Buchler MW, Stathis P and Korc M (1994) Induction and expression of heparin-binding EGF-like growth factor in human pancreatic cancer. *Biochem Biophys Res Commun* **3**: 1705–1709
Ku P and D'Amore PA (1995) Regulation of basic fibroblast growth factor (bFGF) gene and protein expression following its release from sublethally injured endothelial cells. *J Cell Biochem* **58**: 328–343
Laemmli UK (1970) Cleavage of structural proteins during the assembly of the head of bacteriophage T4. *Nature* **227**: 680–685
Lanfrancone L, Borashi D, Ghiara P, Falini B, Grignani F, Peri G, Mantovani A and Pelicci PG (1992) Human peritoneal mesothelial cells produce many cytokines (granulocyte colony-stimulating factor [CSF], granulocyte-monocyte-CSF, macrophage-CSF, interleukin-1 [IL-1], and IL-6) and are activated and stimulated to grow by IL-1. *Blood* **80**: 2835–2842
Melder RJ, Koenig GC, Witwer BP, Safabakhsh N, Munn LL and Jain RK (1996) During angiogenesis, vascular endothelial growth factor and basic fibroblast growth factor regulate natural killer cell adhesion to tumour endothelium. *Nat Med* **2**: 992–997
Miyoshi E, Higashiyama S, Nakagawa T, Hayashi N and Taniguchi N (1997) Membrane anchored heparin-binding epidermal growth factor-like growth factor acts as a tumour survival factor in a hepatoma cell line. *J Biol Chem* **272**: 14349–14355
Montesano R (1992) Regulation of angiogenesis in vitro. *Eur J Clin Invest* **22**: 504–515
Naef M, Yokoyama M, Friess H, Buchler MW and Korc M (1996) Co-expression of heparin-binding EGF-like growth factor and related peptides in human gastric carcinoma. *Int J Cancer* **66**: 315–321
Narita T, Kawakami-Kimura N, Sato M, Matsuura N, Higashiyama S, Taniguchi N and Kannagi R (1996) Alteration of integrins by heparin-binding EGF-like growth factor in human breast cancer cells. *Oncology* **53**: 374–381
Ottow RT, Eggermont AM, Steller EP and Sugarbaker PH (1987) The requirements for successful immunotherapy of intraperitoneal cancer using interleukin-2 and lymphokine activated killer cells. *Cancer* **60**: 1465–1473
Pepper MS, Ferrara N, Orci L and Montesano R (1992) Potent synergism between vascular endothelial growth factor and basic fibroblast growth factor in the induction of angiogenesis in vitro. *Biochem Biophys Res Commun* **189**: 824–831
Radinsky R (1993) Paracrine growth regulation of human colon carcinoma organ-specific metastasis. *Cancer Metastasis Rev* **12**: 345–361
Radinsky R, Risin S, Fan D, Dong Z, Bielenberg D, Bucana CD and Fidler IJ (1995) Level and function of epidermal growth factor receptor predict the metastatic potential of human colon carcinoma cells. *Clin Cancer Res* **1**: 19–31
Rapraeger AC, Krufka A and Olwin BB (1991) Requirement of heparan sulfate for bFGF-mediated fibroblast growth and myoblast differentiation. *Science* **252**: 1705–1708
Rapraeger AC, Guimonds S, Krufka A and Olwin BB (1994) Regulation by heparan sulfate in fibroblast growth factor signaling. *Methods Enzymol* **245**: 219–240
Sato M, Narita T, Kawakami-Kimura N, Higashiyama S, Taniguchi N, Akiyama S, Hashimoto T, Manabe T and Kannagi R (1996) Increased expression of integrins by heparin-binding EGF like growth factor in human esophageal cancer cells. *Cancer Lett* **102**: 183–191
Sironi M, Sciacca FL, Matteucci C, Conni M, Vecchi A, Bernasconi S, Minty A, Caput D, Ferrara P, Colotta F and Mantovani A (1994) Regulation of endothelial and mesothelial cell function by interleukin-13: selective induction of vascular cell adhesion molecule-1 and amplification of interleukin-6 production. *Blood* **84**: 1913–1921
Soker S, Fidder H, Neufeld G and Klagsbrun M (1996) Characterisation of novel vascular endothelial growth factor (VEGF) receptors on tumour cells that bind $VEGF_{165}$ via its exon 7-encoded domain. *J Biol Chem* **271**: 5761–5767
Soker S, Takashima S, Miao HQ, Neufeld G, Klagsbrun M (1998) Neuropilin-1 is expressed by endothelial and tumour cells as an isoform-specific receptor for vascular endothelial growth factor. *Cell* **92**: 735–745
Stylianou E, Jenner LA, Davies M, Coles GA and Williams JD (1990) Isolation, culture and characterisation of human peritoneal mesothelial cells. *Kid Int* **37**: 1563–1570
Tokunaga A, Onda M, Okuda T, Teramoto T, Fujita I, Mizutani T, Kiyama T, Yoshiyuki T, Nishi K and Matsukura N (1995) Clinical significance of epidermal growth factor (EGF), EGF receptor, and c-erbB-2 in human gastric cancer. *Cancer* **75**: 1418–1425
Topley N, MacKenzie RK and Williams JD (1996) Macrophages and mesothelial cells in bacterial peritonitis. *Immunobiology* **195**: 563–573
Ushiro S, Ono M, Izumi H, Kohono K, Taniguchi N, Higashiyama S and Kuwano M (1996) Heparin-binding epidermal growth factor-like growth factor: p91 activation induction of plasminogen activator/inhibitor, and tubular morphogenesis in human microvascular endothelial cells. *Jpn J Cancer Res* **87**: 68–77

*© 2000 Cancer Research Campaign*
